# Predictive ability of the REMS and HOTEL scoring systems for mortality in geriatric patients with pulmonary embolism

**DOI:** 10.1186/s43044-024-00531-0

**Published:** 2024-08-09

**Authors:** Abuzer Özkan, Serdar Özdemir

**Affiliations:** 1 Department of Emergency Medicine, University of Health Sciences Bağcılar Training and Research Hospital, Istanbul, Turkey; 2 Department of Emergency Medicine, University of Health Sciences Ümraniye Training and Research Hospital, Site Mahallesi, Adıvar sokak, No 44/15 Ümraniye, Istanbul, Turkey

**Keywords:** Pulmonary infarction, Geriatrics, HOTEL, Early warning score

## Abstract

**Background:**

Pulmonary embolism (PE) is an important cause of mortality and morbidity in the geriatric population. We aimed to compare the ability of the pulmonary embolism severity index (PESI), rapid emergency medicine score (REMS), and hypotension, oxygen saturation, low temperature, electrocardiogram change, and loss of independence (HOTEL) to predict prognosis and intensive care requirement in geriatric patient with PE.

**Results:**

The median age of 132 patients was 77 (71–82) years. PESI was higher in the non-survivor group [132 (113–172)] (*P* =0.001). The median REMS was 8 (7–10), and it was higher in the non-survivor group [10 (7.5–12.0)] (*p* = 0.005). The median HOTEL score was 1 (0–2) in the whole cohort and 2 (1–3) in the non-survivor group, indicating significant difference compared to the survivor group (*P* = 0.001). The area under the curve (AUC) values of HOTEL, REMS, and PESI were determined as 0.72, 0.65, and 0.71, respectively. For the prediction of intensive care requirement, the AUC values of HOTEL, REMS, and PESI were 0.76, 0.75, and 0.76, respectively, with no significant difference in pairwise comparisons (PESI vs. REMS: *p* = 0.520, HOTEL vs. PESI: *P* = 0.526, REMS vs. HOTEL: *P* = 0.669, overall test: *P* = 0.96, DeLong’s test). The risk ratios of HOTEL and PESI were parallel to each other [5.31 (95% confidence interval (CI): 2.53–11.13) and 5.34 (95% CI: 2.36–12.08), respectively].

**Conclusion:**

HOTEL and REMS were as successful as PESI in predicting short-term mortality and intensive care requirement in geriatric patients with PE. These scores are also more practical since they have fewer parameters than PESI.

## Background

Partial or complete occlusion of the pulmonary arteries is pulmonary embolism (PE). It often occurs as a result of a clot of the lower extremities reaching the lungs. Repeated small embolism or a large embolism can be fatal [1]. It is one of the major causes of in-hospital deaths [2]. The incidence rate of PE is nearly 39–115 persons per 100,000 people. The venous thromboembolism is higher in patients’ elderly over 80 years than in those younger than 50 years [3]. The symptoms of acute PE are non-pathognomonic. There may be hemoptysis, syncope, dyspnea, presyncope, and chest pain [4]. Pulmonary angiography is the gold standard imaging method. According to the European Society of Cardiology (ESC) and the American Heart Association (AHA), PE cases can be divided into three main categories according to severity: low risk (ESC and AHA) with a mean 1-month mortality rate of 1%, intermediate risk (ESC) or submassive (AHA) with a mortality of 2–3% over the 7–30 days follow-up, and high risk (ESC) or massive (AHA) with an approximate 1-month mortality rate of 30% [5].

The prevalence of PE increases with age in elderly patients [6, 7]. In this patient population, the diagnosis of PE presents with difficulties due to the indistinct nature of symptoms, high incidence of PE, and suppression by comorbidities. The rate of short-term mortality reaches 25% in elderly patients. It is known that PE risk classification performs an important function in the management of acute PE in older with multi-morbidities [8].

Scoring systems (SS) are used to assist in the diagnosis, to manage the patient correctly, to predict mortality, and to classify of PE patients according to severity. One of these systems is the pulmonary embolism severity index (PESI), which predicts short-term mortality [4]. PESI consists of 11 parameters and divides patients into five categories according to disease severity. It can prognosticate patients at a rate of 0–24.4% [9].

The hypotension, oxygen saturation, low temperature, electrocardiographic change, and loss of independence (HOTEL) scoring system is used to predict 15–24 h mortality in non-surgical patients [10]. The HOTEL system has similar criteria to PESI but additionally includes an electrocardiography (ECG) evaluation. It is known that there are ECG changes in patients with PE whose hemodynamics is affected [11]. Therefore, it can be assumed that HOTEL can predict mortality and intensive care requirement in patients with PE.

The rapid emergency medicine score (REMS) predicts hospitalization requirement, in-hospital mortality, and length of hospital stay [12, 13]. REMS has been demonstrated to predict mortality in sepsis, trauma, severe acute respiratory syndrome coronavirus 2, and carbon monoxide poisoning [14–16]. The variables included in this score and its predictive success in various patient groups indicate that it can also be used in patients with PE.

In this study, our aim was to assess the skill of HOTEL and REMS to predict short-term mortality and intensive care requirement in geriatric patients with PE.

## Methods

This prospectively designed study was conducted between March 22, 2022, and September 22, 2022, at ******, a tertiary hospital in ****, with a capacity of 646 wards and 166 intensive care beds. At the time of the study, 11 emergency medicine specialists, four emergency medicine academicians, and 32 emergency medicine residents were working in the emergency department (ED).

The study included patients aged over 65 years, with symptoms and signs suggestive of PE, such as chest pain, shortness of breath, hemoptysis, dyspnea, syncope, back pain, fatigue, and pain in the legs. Those who did not volunteer to participate in the study and those whose PE was not confirmed by computed tomography angiography were excluded. Comorbidities, including hypertension, history of malignancy, congestive heart failure, diabetes mellitus, chronic obstructive pulmonary disease, coronary artery disease, chronic kidney disease, cerebrovascular disease, history of surgery, deep vein thrombosis, and conditions requiring anticoagulant use were recorded.

At the time of presentation, the patients’ complaints, pulse rate, carbon dioxide saturation, respiratory rate, body temperature, systolic and diastolic blood pressure, peripheral oxygen saturation, and abnormalities of ECG were recorded. Laboratory parameters and echocardiogram (ECHO) data at presentation and outcomes (intensive care unit or admission to the inpatient ward or discharge from the ED) were also recorded. Written informed consent was obtained from the patients.

At the time of arrival at the ED, the PESI score [17] (gender, age, history of cancer, history of chronic lung disease, history of heart failure, O_2_ saturation < 90%, temperature < 36 °C, heart rate ≥ 110, respiratory rate ≥ 30, and altered mental status) was determined and classified as follows: class I, 1–65; class II, 66–85; class III, 86–105; class IV, 106–125; and class V over 125. The patients in risk classes I and II were defined as having low risk and the remaining patients as having high risk. The REMS (respiratory rate, heart rate, main arterial pressure, age, Glasgow Coma Scale score, and peripheral oxygen saturation) and HOTEL (low temperature, oxygen saturation, hypotension, loss of independence, and ECG changes) scores [18] were also calculated.

The patients were followed for 30 days to determine the outcomes.

Sample size calculation was performed using the G*Power program to ensure accurate and reliable determination of the required sample size. Sample size calculation was performed based on data from a previous study conducted in the same region, yielding similar outcomes [19]. By considering a marginal error of 5%, a type I error rate of 5%, and targeting a sensitivity of 99%, the required sample size was determined to be 124 participants.

Open-source software (Jamovi, Sidney, Australia, https://www.jamovi.org, v. 1.6.21) was utilized for the analysis. The Shapiro–Wilk test was used to the distribution of quantitative variables. Continuous variables as median [interquartile range (IQR)] and categorical variables were expressed as percentages values. The Mann–Whitney *U*-test was conducted to compare quantitative data, since the study groups did not fit the normal distribution. The Chi-square test was used to compare categorical variables. The receiver operating characteristic (ROC) analysis was presented to determine whether the investigated variables could predict short-term mortality and intensive care requirement. The odds ratio values were calculated to determine and compare the predictive ability of the SS. The statistical significance limit was accepted as *P* < 0.05.

The primary outcome was death within 30 days of hospital admission. The secondary outcome was the ability to predict the need for intensive care after initial admission.

## Results

**Fig. 1:**
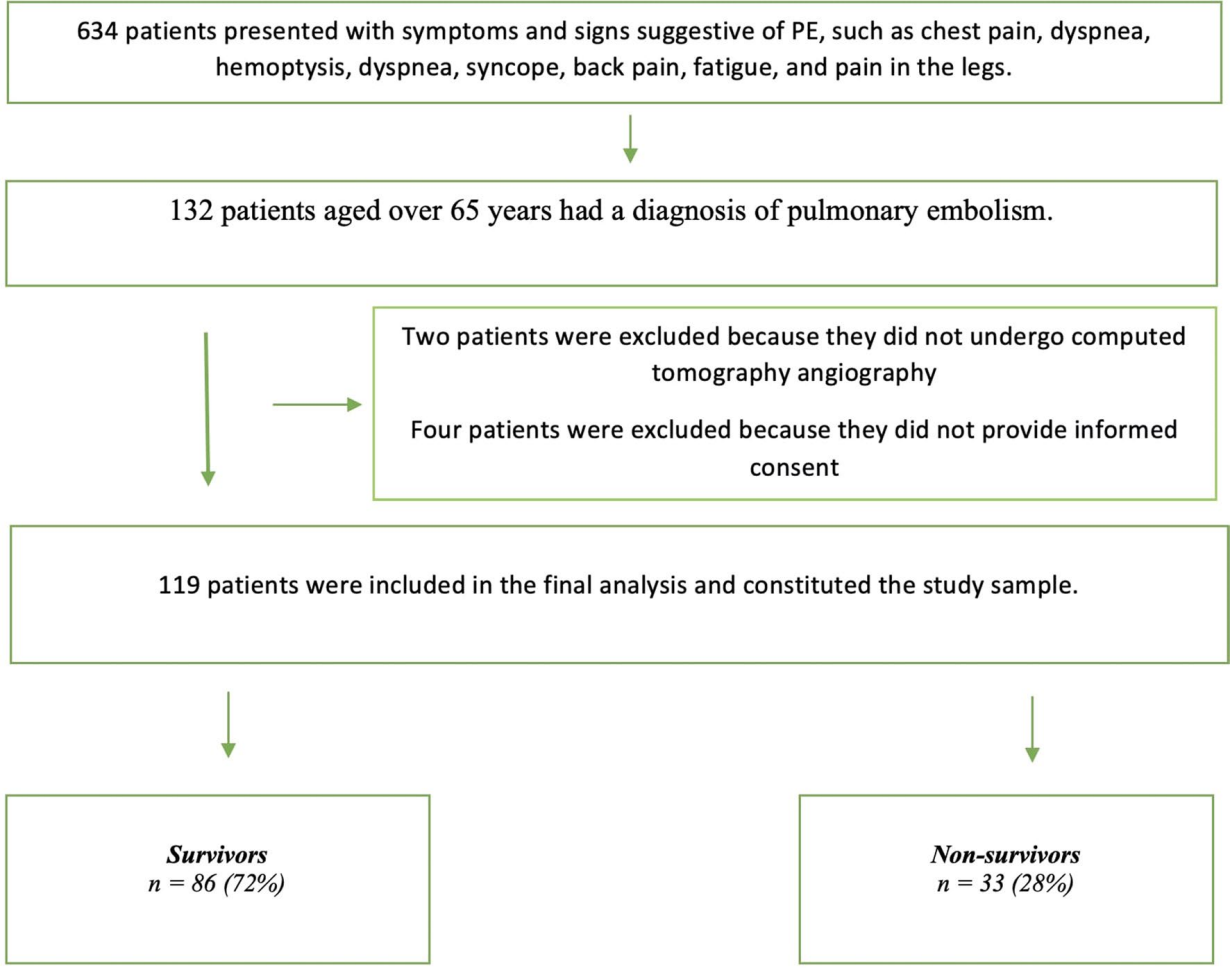
Flowchart of the study

A total of 132 patients over 65 years with PE included in the sample of the study. Four patients with incomplete medical history data and two patients who could not undergo computed tomography pulmonary angiography were excluded. The flowchart of the research is given in Fig. 1. Eventually 119 patients were included in the sample. The median age was 77 (71–82) years. The mortality rate of the sample was 28%. All mortality was in-hospital mortality. In the mortality group, the median age was 78.0 (73.0–84.0) years, and there was no significant difference between the survivor and non-survivor groups (*P* = 0.207). The number of women was 80 (67.2%). The most common complaint of the patients was dyspnea 68 (57.1%). The rate of dyspnea did not result in a significant difference (*p* = 0.990). The most common comorbid disease was hypertension 48 (40.3%). Other comorbidities seen at a high rate in the whole cohort were history of malignancy (*n* = 24, 20.2%), diabetes mellitus (*n* = 21, 17.6%), and cerebrovascular disease (*n* = 21, 17.6%). Thirty-two (26.9%) patients were using anticoagulants.

Mean arterial pressure, oxygen saturation, diastolic blood pressure, and systolic blood pressure were significantly lower in the non-survivor group. The pulse rate was significantly higher in the non-survivor group [115 beats/min (IQR: 93–130, *P* = 0.005)]. The median value of D-dimer was 7110.0 mg/dL (IQR: 3245.0–14540.0) in the whole cohort, 5730.0 mg/dL (IQR: 2835.0–13370.0) in the survivor group, and 13,110.0 mg/dL (IQR: 5712.2–15920.0) in the non-survivor group. The median blood urea nitrogen was 49.2 mg/dl (38.5–68.5) in the whole cohort. This value was significantly higher in the non-survivor group [72.8 mg/dL (42.8–96.3)] (*P* = 0.001). Thrombolytics were administered to 10 (8%) patients. The right ventricular dilatation was the most common ECHO abnormality (*n* = 44, 36%). This was also the most frequently seen ECHO finding in the non-survivor group (*n* = 17, 51%). The highest mortality rate was detected in the group of intensive care unit (*n* = 29, 87.9%).

The median PESI was 116 (89–140). PESI was higher in the non-survivor group [132 (113–172)] (*P* =0.001). The median REMS was 8 (7–10), and it was higher in the non-survivor group [10 (7.5–12.0)] (*p* = 0.005). The median HOTEL score was 1 (0–2) in the whole cohort and 2 (1–3) in the non-survivor group, indicating significant difference compared to the survivor group (*P* = 0.001). Table 1 shows the baseline characteristics of the 119 geriatric patients with PE stratified by mortality status.

**Table 1:** Baseline characteristics of 145 geriatric patients with pulmonary embolism stratified by mortality status

Variables	Total *n* = 119	Survivors *n* = 86 (72%)	Non-survivors *n* = 33 (28%)	*P*
	*n* (%)/median (25th–75th percentiles)	*n* (%)/median (25th–75th percentiles)	*n* (%)/median (25th–75th percentiles)	
Age	77.0 (70.0–82.0)	76.0 (70.0–81.8)	78.0 (73.0–84.0)	0.207
Gender				
Male	39 (32.8)	29 (33.7)	10 (30.3)	0.891
Female	80 (67.2)	57 (66.3)	23 (69.7)	
Symptoms				
Dyspnea	68 (57.1)	49 (57.0)	19 (57.6)	0.990
Chest pain	19 (16.0)	15 (17.4)	4 (12.1)	0.667
Syncope	33 (27.7)	24 (27.9)	9 (27.3)	1.000
Back pain	2 (1.7)	1 (1.2)	1 (3.0)	1.000
Hemoptysis	5 (4.2)	3 (3.5)	2 (6.1)	0.908
Fatigue	26 (21.8)	20 (23.3)	6 (18.2)	0.725
Leg pain	12 (10.1)	8 (9.3)	4 (12.1)	0.907
Comorbidities				
Hypertension	48 (40.3)	34 (39.5)	14 (42.4)	0.937
Chronic obstructive pulmonary disease	14 (11.9)	11 (12.9)	3 (9.1)	0.792
Diabetes mellitus	21 (17.6)	16 (18.6)	5 (15.2)	0.862
Coronary artery disease	20 (16.8)	16 (18.6)	4 (12.1)	0.567
Congestive heart failure	8 (6.7)	4 (4.7)	4 (12.1)	0.295
History of malignancy	24 (20.2)	14 (16.3)	10 (30.3)	0.147
Chronic kidney disease	2 (1.7)	2 (2.3)	0 (0.0)	0.931
Cerebrovascular disease	21 (17.6)	12 (14.0)	9 (27.3)	0.151
History of surgery	17 (14.3)	13 (15.1)	4 (12.1)	0.900
Deep vein thrombosis	11 (9.2)	7 (8.1)	4 (12.1)	0.751
Anticoagulant use	32 (26.9)	19 (22.1)	13 (39.4)	0.094
Vital parameters				
Systolic blood pressure mm/hg	123 (103.5–144.5)	125.5 (110–156.8)	105 (95–130)	0.001
Diastolic blood pressure	74 (61.5–87)	80 (66.2–90)	67 (55–80)	0.01
Pulse rate	100 (87–120)	100 (83.5–109.8)	115 (93–130)	0.005
Oxygen saturation	90 (85–95)	91 (87–95)	86 (82–90)	< 0.001
Carbon dioxide saturation	37.8 (31.5–43.5)	37 (30.6–43)	40.2 (33.0–46.3)	0.092
Fever	36.4 (36–36.8)	36.4 (36.0–36.7)	36.6 (36–37)	0.079
Respiratory rate	19 (17.5–22.5)	19 (17.2–21)	21 (18–25)	0.141
Mean arterial pressure	91.3 (77–107.5)	93.3 (82.3–111.1)	81.7 (69.7–94)	0.004
Laboratory parameters				
White blood cell count (10^3^/µL)	10.8 (8.7–13.7)	10.6 (8.5–13)	11.3 (9.3–14.2)	0.321
pH	7.4 (7.4–7.5)	7.4 (7.4–7.5)	7.4 (7.3–7.4)	0.039
Hemoglobin (g/dL)	12.5 (11–13.3)	12.6 (11.2–13.3)	12.2 (10.2–13.3)	0.201
Hematocrit (%)	38.4 (33.6–41)	38.4 (34.3–41)	38.3 (32.1–40.8)	0.463
Blood urea nitrogen (mg/dL)	49.2 (38.5–68.5)	47.1 (36.9–53.5)	72.8 (42.8–96.3)	< 0.001
Creatinine (mg/dL)	1.0 (0.8–1.2)	1.0 (0.8–1.2)	1.0 (0.8–1.1)	0.404
Sodium (mEq/L)	138.0 (134–140)	138.0 (135.0–140)	137.0 (133.0–144)	0.493
Potassium (mmol/L)	4.3 (4–4.8)	4.3 (4.0–4.8)	4.2 (4–4.8)	0.936
D-dimer (mg/dL)	7110 (3245–14540)	5730 (2835–13370)	13110 (5712.2–15920)	0.254
Glucose (mg/dL)	143.0 (116.8–196.8)	143 (118–189)	151 (116–220.5)	0.845
Electrocardiography finding				
Normal sinus rhythm	61 (51.3%)	50 (58.1%)	11 (33.3%)	0.015
ST depression	2 (1.7%)	0 (0%)	2 (6.1%)	0.021
Sinus tachycardia	32 (26.9%)	20 (23.3%)	12 (36.4%)	0.149
Atrial fibrillation	13 (10.9%)	6 (7.0%)	7 (21.2%)	0.026
T negativity	4 (3.4%)	3 (3.5%)	1 (3.0%)	0.901
Left bundle branch block	1 (0.8%)	1 (1.2%)	0 (0%)	0.534
Right bundle branch block	3 (2.5%)	3 (3.5%)	0 (0%)	0.277
S1Q3T3	3 (2.5%)	3 (3.5%)	0 (0%)	0.277
Echocardiogram finding				
Normal	21 (18%)	16 (19%)	5 (15%)	0.504
Right ventricular dilatation	44 (36%)	28(32%)	17 (51%)	0.190
Elevated PAP	8 (7%)	7 (8%)	1 (3%)	0.266
Not evaluated	41			
Outcome				
Discharge	3 (2.5)	3 (3.5)	0 (0)	0.665
Admission—inpatient ward	79 (66.4)	75 (87.2)	4 (12)	< 0.001
Admission—intensive care unit	37 (31.1)	8 (9.3)	29 (87.9)	< 0.001
Scores				
PESI	116 (89–140)	107.5 (85.2–128)	132 (112.5–171.5)	< 0.001
REMS	8 (7–10)	8 (7–10)	10 (7.5–12)	0.005
HOTEL	1 (0–2)	1 (0–2)	2 (1.0–3)	< 0.001

Abbreviations: Rapid emergency medicine score, REMS; pulmonary embolism severity index, PESI; hypotension, oxygen saturation, low temperature, electrocardiogram change, and loss of independence, HOTEL; and pulmonary artery pressure, PAP

**Fig. 2:**
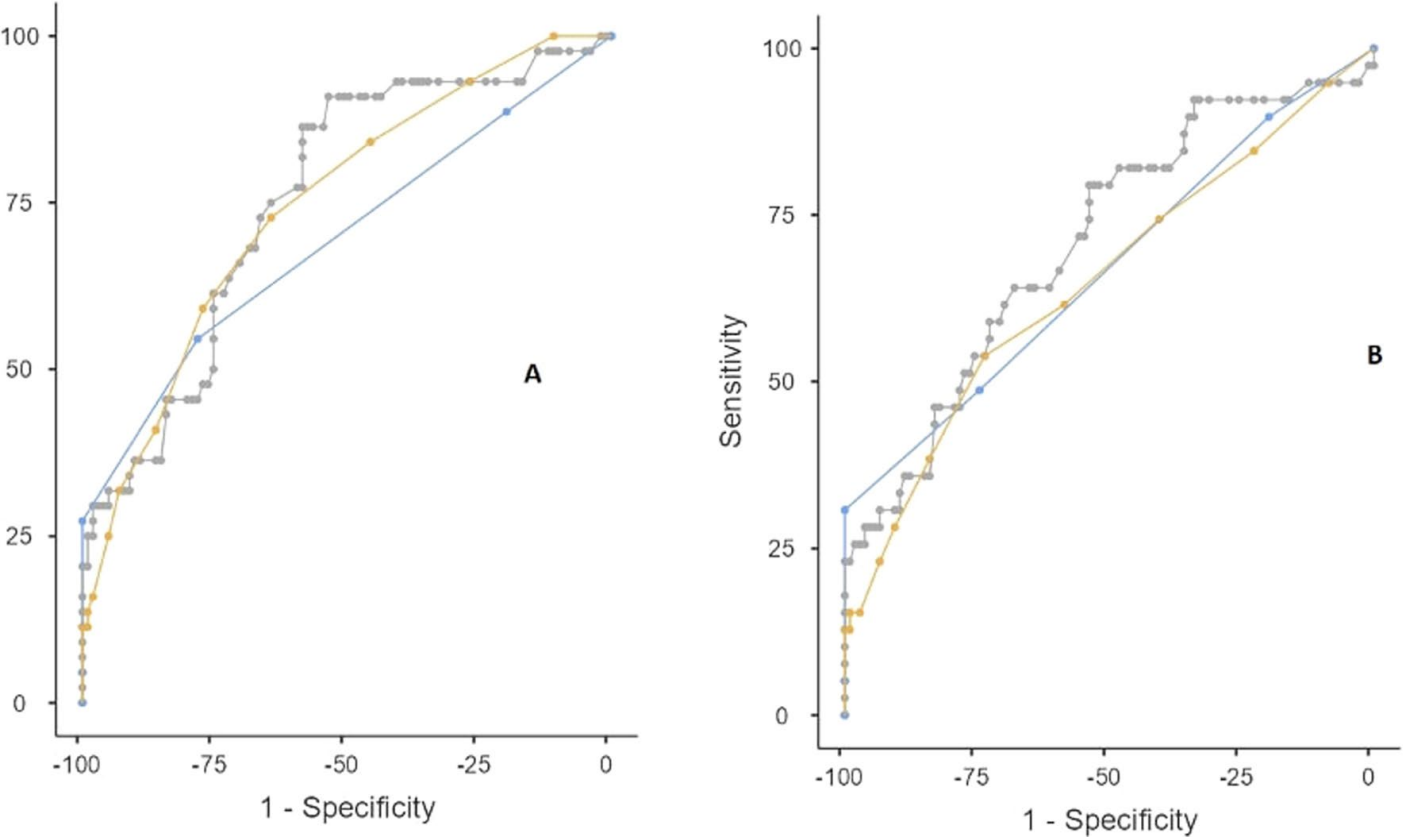
Area under the receiver operating characteristic curve values of the investigated scoring systems in the prediction of intensive care requirement **a** and short-term mortality **b**

The area under the curve (AUC) values of HOTEL, REMS, and PESI were determined as 0.72, 0.65, and 0.71, respectively. For the prediction of intensive care requirement, the AUC values of HOTEL, REMS, and PESI were 0.76, 0.75, and 0.76, respectively, with no significant difference in pairwise comparisons (PESI vs. REMS: *p* = 0.520, HOTEL vs. PESI: *P* = 0.526, REMS vs. HOTEL: *P* = 0.669, overall test: *P* = 0.96, DeLong’s test) (Table 2, Fig. 2). The risk ratios of HOTEL and PESI were parallel to each other [5.31 (95% confidence interval (CI): 2.53–11.13) and 5.34 (95% CI: 2.36–12.08), respectively] (Table 3).

**Table 2:** AUC values of the investigated scoring systems in the prediction of short-term mortality and intensive care requirement

	Cutoff	Sensitivity (%)	Specificity (%)	PPV (%)	NPV (%)	Youden's index	AUC
Short-term mortality							
HOTEL	2	66.67	72.64	47.27	85.56	0.39	0.72
REMS	10	53.85	73.58	42.86	81.25	0.27	0.65
PESI	111	79.49	53.77	38.75	87.69	0.33	0.71
Intensive care requirement							
HOTEL	2	65.91	74.26	52.73	83.33	0.4	0.76
REMS	9	72.73	64.36	47.06	84.42	0.37	0.75
PESI	111	86.36	58.42	47.50	90.77	0.45	0.76

Abbreviations: Rapid emergency medicine score, REMS; pulmonary embolism severity index, PESI; hypotension, oxygen saturation, low temperature, electrocardiogram change, and loss of independence, HOTEL; area under the curve, AUC; positive predictive value, PPV; and negative predictive value, NPV

**Table 3:** Odds ratio and 95% confidence interval values of the scoring systems

	Risk ratio	95% confidence intervals		*p*
		Lower bound	Upper bound	
Short-term mortality				
PESI	5.34	2.36	12.08	< 0.001
REMS	2.92	1.43	5.97	0.003
HOTEL	5.31	2.53	11.13	< 0.001
Intensive care requirement				
PESI	8.9	3.45	22.95	< 0.001
REMS	4.9	2.29	10.47	< 0.001
HOTEL	5.58	2.59	12.00	< 0.001

Abbreviations: Rapid emergency medicine score, REMS; pulmonary embolism severity index, PESI; and hypotension, oxygen saturation, low temperature, electrocardiogram change, and loss of independence, HOTEL

## Discussion

In our study, we evaluated 119 geriatric patients with PE over 65 years of age. It is known that PESI predicts short-term mortality, which was also validated in our study. At the same time, according to our results, the REMS and HOTEL scores predicted 30 days mortality in geriatric patients with PE as significantly and strongly as PESI. Furthermore, both scores were as powerful as PESI in predicting intensive care requirement in these patients. To the authors’ knowledge, this is the first research to evaluate the prognostic value of HOTEL and REMS in geriatric PE.

In the ED, the workload is intense, and there is a race against time. Therefore, it is crucial that all evaluations provide accurate results and take a short time. SS have thus been developed to support or exclude a diagnosis in patients requiring intensive intervention, such as those with PE [20]. These scores also assist clinicians in determining the severity of emergency cases, refer the patient to the right unit in the post-emergency period, and ensure the efficient use of resources [21].

PESI is a widely used scoring system for PE in all age groups. It consists of 11 independent predictors of disease and clinical state [22]. Although this increases the power of PESI to predict mortality, it also creates a limitation. Three of the parameters included in PESI (history of chronic lung disease, history of cancer, and history of heart failure) are based on anamnesis information. This means that PESI cannot be used in patients whose consciousness level is not sufficient or whose disease history is unknown. This is a major problem in the use of PESI. Aujesky et al. did not make any suggestions to eliminate this limitation when presenting this scoring system [17]. The HOTEL and REMS scores, on the other hand, include some parameters of PESI and are completely based on clinical data. Thus, the consciousness level of the patient or lack of anamnesis is no longer a major problem. There are six parameters in REMS and five parameters in the HOTEL scoring system [23]. Therefore, another advantage of both scores compared to PESI is that they contain fewer parameters. In parallel with this claim, Aujesky et al. also suggested using a simplified version of PESI, which consists of six parameters because it is easier to use. These advantages of HOTEL and REMS, combined with their success in predicting mortality and intensive care requirement, show that they are important potential alternatives to PESI.

In the original study of PESI, Aujesky et al. found the AUC value to be 0.78 (95% CI: 0.77–0.80) in predicting 30 days mortality in patients with PE [17]. In a later meta-analysis, they reported that PESI had an AUC value of 0.7853 (standard deviation: 0.0058) in predicting overall weighted mortality [9]. Despite the small sample size in our study, the AUC values of the three SS were similar in predicting intensive care requirement and mortality. According to our outcomes, the cut-off value of PESI was 111 points, and mortality significantly increased in the patients who scored above this value. Similarly, in the original PESI study including 15,531 patients, this value was reported in high-risk patients (classes III–V) [17].

APACHE II is a well-known scoring system used in the management of critically ill patients. The REMS score, a simplified and easily calculable scoring system, is derived from the more complex APACHE II system [24–27]. REMS was originally planned to predict the risk of in-hospital mortality [24]. Subsequent studies have shown that REMS strongly predicted poor outcomes in critically ill patients in the ED. In a systematic review, Ghaffarzad et al. reported that REMS successfully predicted 30-day mortality with an AUC value of 0.79 [28]. In another study conducted in the ED with Asian patients, Ha et al. evaluated 1746 non-trauma causes and reported the median REMS score as 6 (IQR: 5–8) and the AUC value as 0.712 for the prediction of 30 days mortality [29]. In a sample including 225 patients with sepsis, Chatchumni et al. observed that REMS had an AUC value of 0.886 and, therefore, suggested that this parameter could be an important predictor of mortality [15]. Our study showed that, REMS had a similar predictive power in predicting mortality. We determined the median REMS as 10, and the AUC value was 0.650. Our slightly lower AUC value can be attributed to our smaller sample size. However, REMS showed better performance in predicting intensive care requirement in our study. Both previous studies and our results suggest that REMS can predict 30 days mortality and intensive care requirement in critically ill patients, such as geriatric patients with PE.

In a previous study, the HOTEL score was reported to be successful in predicting early mortality (15 min–24 h) [18]. Stræde et al. also showed that the HOTEL score had excellent performance in predicting short-term mortality in 1576 medical patients [10]. In another study conducted with 939 geriatric patients in the ED, Dündar et al. found that the HOTEL score strongly predicted intensive care requirement and in-hospital mortality [23]. In the current study, the HOTEL score was as powerful as PESI in predicting short-term mortality and intensive care requirement. Although the HOTEL scoring system was not developed for PE, the parameters it contains are relevant for poor outcomes in this condition. At the same time, the HOTEL score consists of fewer parameters than PESI, which makes it more practical for clinical use in patients with PE. In addition, in the HOTEL score, ECG findings other than a normal sinus rhythm, tachycardia, or bradycardia are considered abnormal. They showed ECG changes in patients with PE in the presence of lobar artery or remote branch and pulmonary trunk involvement [30]. The authors also suggested that ECG would assist clinicians in risk stratification and administration of treatment. Although PESI has 11 parameters, we consider that the similar predictive power of the HOTEL scoring system with only five parameters is due to the inclusion of an ECG evaluation.

Among the patients, 2.5% were discharged, 66.4% were admitted to the inpatient ward, and 31.1% were admitted to the intensive care unit. None of the discharged patients died within 30 days. All mortality was in-hospital mortality. Notably, intensive care unit admission was significantly higher among non-survivors (87.9%) compared to survivors (9.3%), for both inpatient ward and intensive care unit admissions. These results highlight the effectiveness of the scoring system in clinical decision-making, particularly in determining the necessity for intensive care unit admission and risk of death. The significant difference in intensive care unit admissions between survivors and non-survivors underscores the scoring system's ability to accurately predict severe cases requiring intensive care.

This study had certain limitations. The sample size was small. Although this did not prevent significant results, we consider that stronger results can be obtained in larger groups. Second, we were not able to determine whether the abnormal ECG findings existed in the pre-embolism period or developed due to PE. Therefore, all the ECGs with abnormal findings (except sinus tachycardia and sinus bradycardia) were considered abnormal.

## Conclusions

In conclusion, HOTEL and REMS were as successful as PESI in predicting short-term mortality and intensive care requirement in geriatric patients with PE. These scores are also more practical since they have fewer parameters than PESI. Considering the intense patient load of the ED, in order to ensure the efficient use of resources, we suggest that the HOTEL and REMS scoring systems can be used instead of PESI to predict mortality and intensive care requirement in geriatric patients with PESI.

## Data Availability

All data generated or analyzed during this study are included in this published article.

## Abbreviations

PE: Pulmonary embolism
PESI: Pulmonary embolism severity index
REMS: Rapid emergency medicine score
HOTEL: Hypotension, oxygen saturation, low temperature, electrocardiogram change, and loss of independence
AUC: Area under the curve
CI: Confidence interval
ESC: European Society of Cardiology
AHA: American Heart Association
SS: Scoring systems
ECG: Electrocardiography
ED: Emergency department
IQR: Interquartile range
ROC: Receiver operating characteristic
ECHO: Echocardiography
